# Rumphellaoic Acid A, a Novel Sesquiterpenoid from the Formosan Gorgonian Coral *Rumphella antipathies*

**DOI:** 10.3390/md12125856

**Published:** 2014-12-04

**Authors:** Hsu-Ming Chung, Wei-Hsien Wang, Tsong-Long Hwang, Lee-Shing Fang, Zhi-Hong Wen, Jih-Jung Chen, Yang-Chang Wu, Ping-Jyun Sung

**Affiliations:** 1 Department of Applied Chemistry, National Pingtung University, Pingtung 900, Taiwan; E-Mail: shiuanmin@mail.npue.edu.tw; 2 Department of Marine Biotechnology and Resources, Asia-Pacific Ocean Research Center, National Sun Yat-sen University, Kaohsiung 804, Taiwan; E-Mails: whw@mail.nsysu.edu.tw (W.-H.W.); wzh@mail.nsysu.edu.tw (Z.-H.W.); 3 National Museum of Marine Biology and Aquarium, Pingtung 944, Taiwan; 4 Graduate Institute of Natural Products, School of Traditional Medicine, College of Medicine, and Chinese Herbal Medicine Research Team, Healthy Aging Research Center, Chang Gung University, Taoyuan 333, Taiwan; E-Mail: htl@mail.cgu.edu.tw; 5 Department of Cosmetic Science and Research Center for Industry of Human Ecology, Chang Gung University of Science and Technology, Taoyuan 333, Taiwan; 6 Department of Sport, Health and Leisure, Cheng Shiu University, Kaohsiung 833, Taiwan; E-Mail: lsfang@csu.edu.tw; 7 Department of Pharmacy, Tajen University, Pingtung 907, Taiwan; E-Mail: jjchen@tajen.edu.tw; 8 School of Pharmacy, College of Pharmacy, China Medical University, Taichung 404, Taiwan; 9 Chinese Medicine Research and Development Center, China Medical University Hospital, Taichung 404, Taiwan; 10 Center for Molecular Medicine, China Medical University Hospital, Taichung 404, Taiwan; 11 Graduate Institute of Marine Biology, Department of Life Science and Institute of Biotechnology, National Dong Hwa University, Pingtung 944, Taiwan; 12 Graduate Institute of Natural Products, Kaohsiung Medical University, Kaohsiung 807, Taiwan

**Keywords:** *Rumphella antipathies*, gorgonian, rumphellaoic acid, sesquiterpenoid, elastase

## Abstract

A novel sesquiterpenoid, rumphellaoic acid A (**1**), was isolated from the gorgonian coral *Rumphella antipathies*, and was found to possess a carbon skeleton that was obtained for the first time from a natural sources. The structure of **1** was elucidated by spectroscopic methods and this compound and was found to exert a moderate inhibitory effect on the release of elastase by human neutrophils.

## 1. Introduction

**Chart 1:**
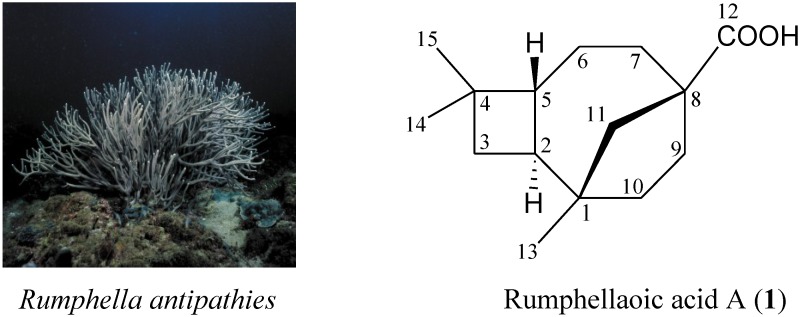
The gorgonian *Rumphella antipathies* and the structure of rumphellaoic acid A (**1**).

Sesquiterpenoid analogs, particularly caryophyllane- and clovane-type analogs, are major constituents of the extracts of gorgonian coral *Rumphella antipathies* [1,2,3,4,5,6,7,8,9,10,11,12,13,14,15,16]. Our continuing studies on the chemical constituents of *R. antipathies* (family Gorgoniidae) (Chart 1), collected off the waters of Taiwan, have led to the isolation of a novel sesquiterpenoid, rumphellaoic acid A (**1**) (Chart 1 and Supplementary Figures S1–S7).

## 2. Results and Discussion

Rumphellaoic acid A (**1**), ${\left[\text{α}\right]}_{\text{D}}^{23}$ −32 (*c* 0.07, CHCl_3_), was isolated as a colorless oil that gave a protonated molecule [M + H]^+^ at *m/z* 237.1832 in the high resolution electronspray ionization mass spectrum (HRESIMS), indicating the molecular formula C_15_H_24_O_2_ (calcd for C_15_H_24_O_2_ + H^+^, 237.1849) and implying four degrees of unsaturation. Comparison of the ^1^H NMR (Table 1) and distortionless enhancement by polarization transfer (DEPT) spectral data with the molecular formula indicated that there must be an exchangeable proton, and this deduction was supported by a broad absorption at 2500–3200 cm^–1^ and a strong absorption at 1696 cm^–1^ for a carboxyl group in the IR spectrum. From the heteronuclear multiple-bond correlation (HMBC) spectrum of **1** (Table 1), a carbonyl resonance at δ_C_ 184.2 (C-12) confirmed the presence of a carboxyl group in **1**. Therefore, from the NMR data, a degree of unsaturation was accounted for and **1** must be a tricyclic compound. In addition, three methyl singlets (H_3_-13, H_3_-14 and H_3_-15), two aliphatic methine protons (H-2 and H-5) and six pairs of aliphatic methylene protons (H_2_-3, H_2_-6, H_2_-7, H_2_-9, H_2_-10 and H_2_-11) were observed in the ^1^H NMR and heteronuclear multiple-quantum coherence (HMQC) spectrum of **1**.

**Table 1:** ^1^H and ^13^C NMR data, ^1^H–^1^H correlation spectroscopy (COSY) and HMBC correlations for sesquiterpenoid **1**.

Position	δ_H_ (*J* in Hz)	δ_C_, Multiple	^1^H-^1^H COSY	HMBC
1		41.9, C		
2	1.58 m	46.0, CH	H_2_-3, H-5	C-1, -6, -10, -13
3α	1.56 m	37.2, CH_2_	H-2, H-3β	C-1, -2
β	1.35 m		H-2, H-3α	C-2, -4, -14, -15
4		33.5, C		
5	1.55 m	48.9, CH	H-2, H_2_-6	C-2, -14
6a	1.32 m	25.8, CH_2_	H-5, H-6b, H_2_-7	C-2, -4, -7
b	1.64 m		H-5, H-6a, H_2_-7	C -2, -4, -5, -7, -8
7a	1.61 m	34.2, CH_2_	H_2_-6, H-7b	C-5, -6, -8, -9, -12
b	1.78 dd (12.8, 5.6)		H_2_-6, H-7a	C-5, -8
8		52.6, C		
9a	1.68 m	29.4, CH_2_	H-9b, H_2_-10	C-7, -8, -10, -11
b	2.14 dd (9.6, 7.6)		H-9a, H_2_-10	C-1, -8, -10, -11, -12
10a	1.44 m	45.4, CH_2_	H_2_-9, H-10b	n.o. ^a^
b	1.68 m		H_2_-9, H-10a	C-8, -9, -11
11α	1.59 d (12.8)	48.9, CH_2_	H-11β	C-1, -2, -7, -8, -9, -10, -12
β	1.94 dd (12.8, 2.4)		H-11α	C-9, -10
12		184.2, C		
13	0.93 s	22.0, CH_3_		C-1, -2, -10, -11
14	0.98 s	20.3, CH_3_		C-3, -4, -5, -15
15	0.98 s	30.5, CH_3_		C-3, -4, -5, -14

^a^ n.o. = not observed.

The gross structure of **1** and all ^1^H and ^13^C NMR data associated with the molecule were determined and verified by 2D NMR studies. ^1^H NMR coupling information in the ^1^H-^1^H COSY spectrum of **1** enabled identification of C2-C5-C6-C7 and C9-C10 (Table 1). These data, together with the HMBC correlations between H-2/C-1, -6, -10; H-5/C-2; H_2_-6/C-2, -5, -7, -8; H_2_-7/C-5, -6, -8, -9; H_2_-9/C-1, -7, -8, -10; and H_2_-10/C-8, -9, established the connectivity within the eight-membered ring (Table 1). The cyclobutane ring, which is fused to the eight-membered ring at C-2 and C-5, was established by the ^1^H-^1^H COSY correlation between H-2 and H_2_-3, and by the HMBC correlations between H_2_-6/C-4 and H_2_-3/C-1, -2. The two tertiary methyls at C-4 were elucidated by the HMBC correlations between H_3_-14/C-3, -4, -5, -15 and H_3_-15/C-3, -4, -5, -14. Moreover, the tertiary methyl at C-1 was confirmed by the HMBC correlations between H_3_-13/C-1, -2, -10, -11. The presence of a carboxyl group at C-8 was deduced from the HMBC correlations between the C-7, C-9 and C-11 methylene protons and the carbonyl carbon of the carboxyl group at δ_C_ 184.2 (C-12). The C-11 methylene bridge between C-1 and C-8 was linked by the HMBC correlations between H_2_-9, H-10a, H_3_-13/C-11; H-11α/C-1, -2, -7, -8, -9, -10, -12; and H-11β/C-9, -10. Based on the above observations, the planar structure of **1** was elucidated unambiguously.

**Figure 1:**
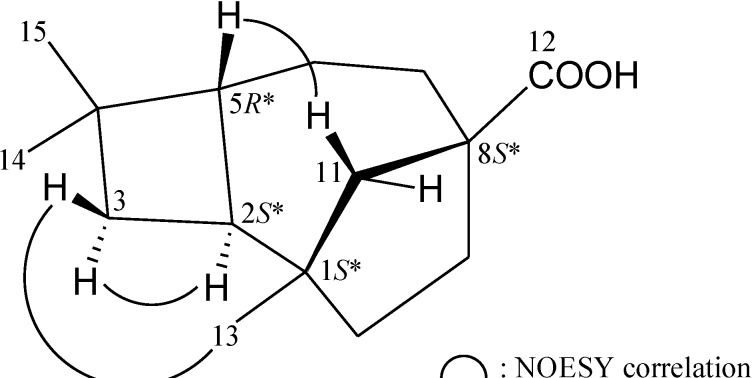
Selective key NOESY correlations of **1**.

The relative configuration of **1** was established from the interactions observed in nuclear Overhauser effect spectroscopy (NOESY) spectra (Figure 1). In the NOESY spectra of **1**, the correlation of H-5 with one proton of the C-11 methylene (δ_H_ 1.94), but not with H-2, indicated that these protons were situated on the same face, and these were assigned as β protons, since H-2 is α-substituted at C-2. It was found that one of the methylene protons at C-3 (δ_H_ 1.56) exhibited a correlation with H-2, and therefore it was assigned as H-3α, and the other C-3 proton (δ_H_ 1.35) as H-3β. The C-13 methyl showed a correlation with H-3β, but not with H-3α, demonstrating that the C-1 chiral carbon possesses an S*-configuration. Furthermore, the carboxyl group at C-8 was proven to possess an S*-configuration by modeling analysis. Based on the above findings, the structure of **1** was elucidated and the chiral carbons for **1** were assigned as 1S*, 2S*, 5R* and 8S*.

It is worth noting that a sesquiterpenoid analog possessing the carbon skeleton descried for **1** was obtained from a natural source for the first time in this study. The *in vitro* anti-inflammatory effect of **1** was tested and this compound was found to display a modest inhibitory effect on the release of elastase (inhibition rate = 29.2%) by human neutrophils at a concentration of 10 μg/mL.

## 3. Experimental Section

### 3.1. General Experimental Procedures

Optical rotation values were measured with a Jasco P-1010 digital polarimeter (Japan Spectroscopic Corporation, Tokyo, Japan). IR spectra were obtained on a Varian Digilab FTS 1000 FT-IR spectrophotometer (Varian Inc., Palo Alto, CA, USA); peaks are reported in cm^−1^. NMR spectra were recorded on a Varian Mercury Plus 400 NMR spectrometer (Varian Inc., Palo Alto, CA, USA) using the residual CHCl_3_ signal (δ_H_ 7.26 ppm) as the internal standard for ^1^H NMR and CDCl_3_ (δ_C_ 77.1 ppm) for ^13^C NMR. Coupling constants (*J*) are given in Hz. ESIMS and HRESIMS were recorded using a Bruker 7 Tesla solariX FTMS system (Bruker, Bremen, Germany). Column chromatography was performed on silica gel (230–400 mesh, Merck, Darmstadt, Germany). TLC was carried out on precoated Kieselgel 60 F_254_ (0.25 mm, Merck, Darmstadt, Germany); spots were visualized by spraying with 10% H_2_SO_4_ solution followed by heating. Normal-phase HPLC (NP-HPLC) was performed using a system comprised of a Hitachi L-7110 pump (Hitachi Ltd., Tokyo, Japan), a Hitachi L-7455 photodiode array detector (Hitachi Ltd., Tokyo, Japan) and a Rheodyne 7725 injection port (Rheodyne LLC, Rohnert Park, CA, USA). A semi-preparative normal-phase column (Hibar 250 × 10 mm, LiChrospher Si 60, 5 μm, Merck, Darmstadt, Germany) was used for HPLC.

### 3.2. Animal Material

Specimens of the gorgonian coral *Rumphella antipathies* (Nutting) were collected by hand using scuba equipment off the coast of Pingtung, Southern Taiwan. This organism was identified by comparison with previous descriptions [17]. A voucher specimen (Specimen No. NMMBA-TWGC-010) was deposited in the National Museum of Marine Biology and Aquarium, Taiwan.

### 3.3. Extraction and Isolation

Sliced bodies of the gorgonian *R. antipathies* (wet weight 402 g, dry weight 144 g) were extracted with a mixture of methanol (MeOH) and dichloromethane (CH_2_Cl_2_) (1:1) at room temperature. The extract was partitioned with ethyl acetate (EtOAc) and H_2_O. The EtOAc layer was separated by silica gel and eluted using *n*-hexane/EtOAc (stepwise, 25:1–pure EtOAc) to yield 29 fractions. Every fraction was checked using the ^1^H NMR spectra. Fraction 15 was re-purified by normal-phase HPLC (NP-HPLC) using a mixture of *n*-hexane and EtOAc as the mobile phase to afford **1** (1.6 mg, 5:1).

Rumphellaoic acid A (**1**): Colorless oil; ${\left[\text{α}\right]}_{\text{D}}^{23}$ −32 (*c* 0.07, CHCl_3_); IR (neat) ν_max_ 2500–3200 (broad), 1696 cm^−1^; ^1^H (400 MHz, CDCl_3_) and ^13^C (100 MHz, CDCl_3_) NMR data, see Table 1; ESIMS: *m/z* 237 [M + H]^+^; HRESIMS: *m/z* 237.1832 (calcd for C_15_H_24_O_2_ + H^+^, 237.1849).

### 3.4. Human Neutrophil Elastase Release

Human neutrophils were obtained by means of dextran sedimentation and Ficoll centrifugation. Briefly, elastase release experiments were performed using MeO-Suc-Ala-Ala-Pro-Val-*p*-nitroanilide as the elastase substrate [18,19]. Elastatinal was used as a reference compound in the anti-inflammatory test of the inhibitory effects on the release of elastase (IC_50_ = 60.0 μM) by human neutrophils in response to fMet-Leu-Phe/Cytochalastin B (fMLP/CB). In the *in vitro* anti-inflammatory bioassay, the inhibitory effects on the release of elastase by activated neutrophils were used as indicators. At a concentration of 10 μg/mL, for the significant activity of pure compounds, an inhibition rate ≥50% is required (inhibition rate ≤ 10%, not active; 20% ≥ inhibition rate ≥ 10%, weakly anti-inflammatory; 50% ≥ inhibition rate ≥ 20%, modestly anti-inflammatory).

## 4. Conclusions

In continuing studies of new substances from marine invertebrates collected off the waters of Taiwan, a new sesquiterpenoid, rumphellaoic acid A (**1**), was isolated from *R. antipathies*. The structure of sesquiterpenoid **1** was elucidated on the basis of spectroscopic methods, and this compound was found to display an inhibitory effect on the release of elastase by human neutrophils. The sesquiterpenoid analogues prepared by chemical methods and biotransformation by Collado’s group [20,21,22] possessed the same carbon skeleton as that of **1**. However, to the best of our knowledge, this is the first time that compound **1** has been obtained from a natural source.

## Supplementary Files

Supplementary File 1
