# Supplementary material for: Rumphellaoic Acid A, a Novel Sesquiterpenoid from the Formosan Gorgonian Coral Rumphella antipathies
Source: Mar Drugs. 2014 Dec 4;12(12):5856–63. doi: 10.3390/md12125856 (PMC4278205; doi:10.3390/md12125856)
Supplement: Supplementary File 1 [file marinedrugs-12-05856-s001.pdf]

Supplementary Information

Figure S1. ^{1}H NMR spectrum (400 MHz) of compound **1** in $CDCl_3$.

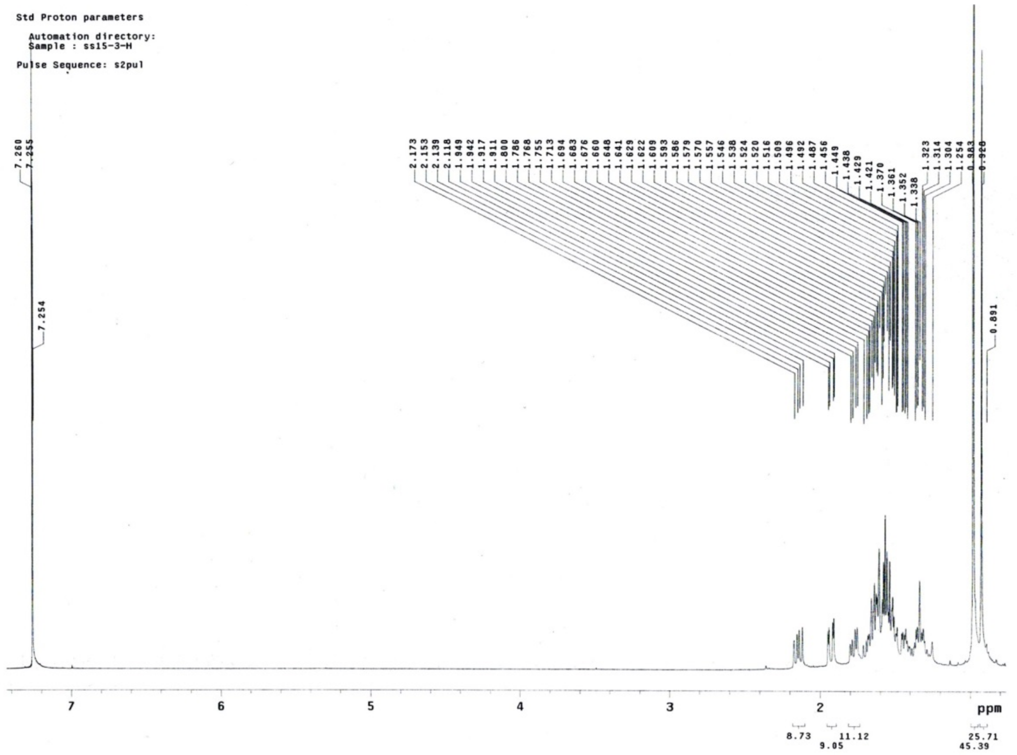

Figure S2. ^{13}C NMR spectrum (100 MHz) of compound **1** in $CDCl_3$.

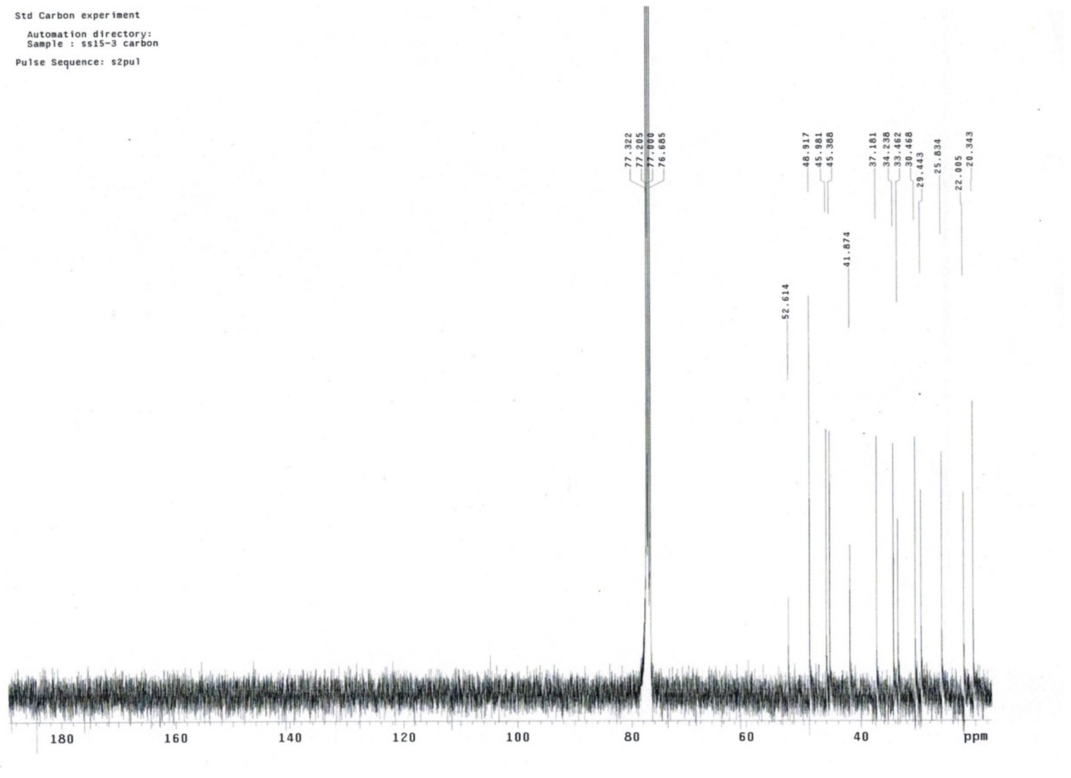

Figure S3. HMQC spectrum (400 MHz) of compound **1** in $CDCl_3$.

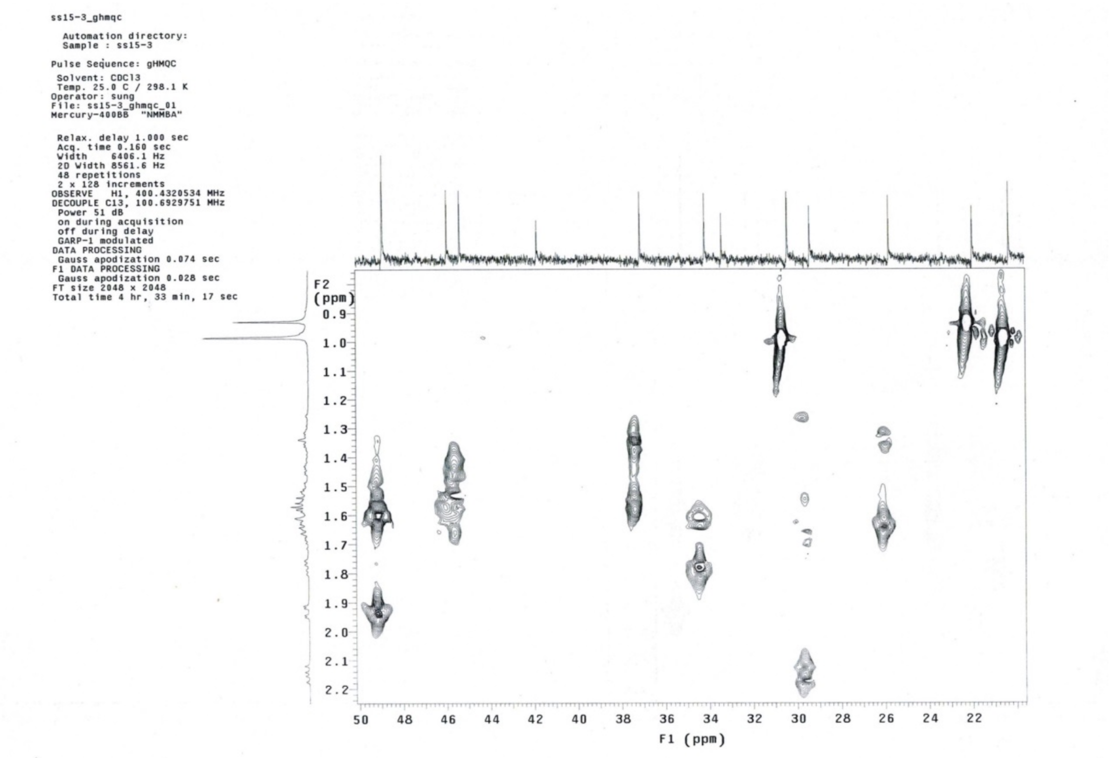

Figure S4. HMBC spectrum (400 MHz) of compound **1** in $CDCl_3$.

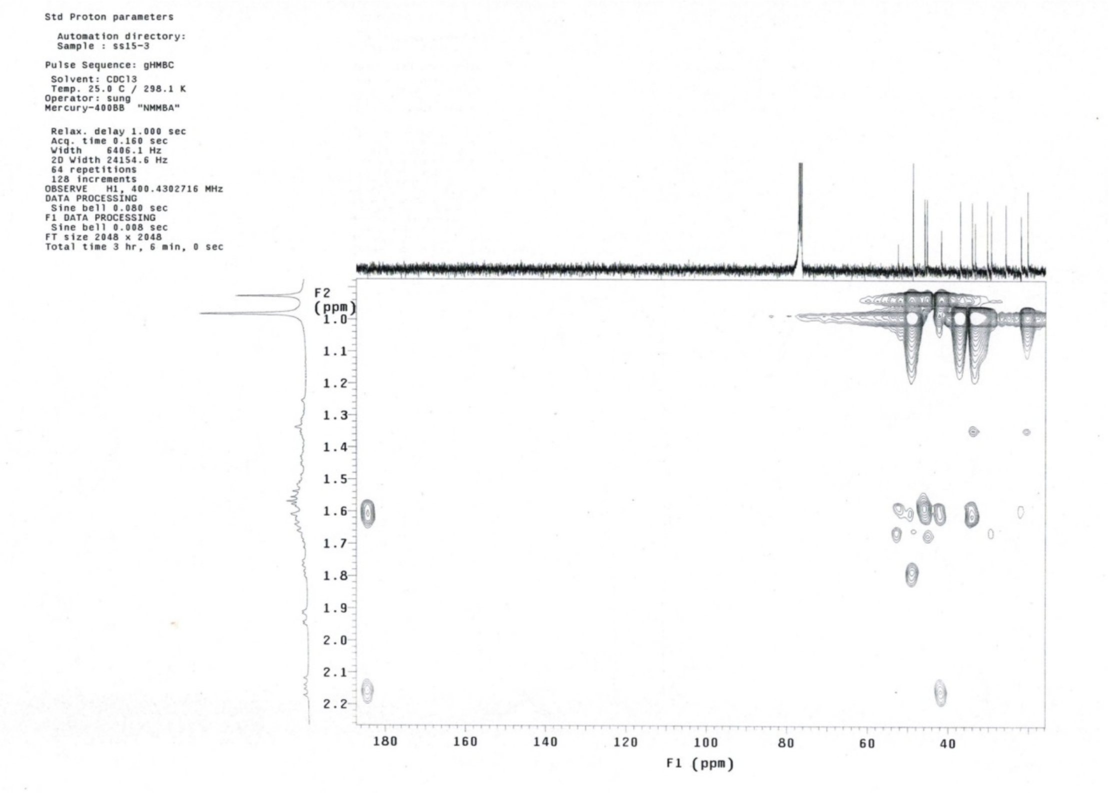

Figure S5. HMBC spectrum (400 MHz) of compound **1** in $CDCl_3$.

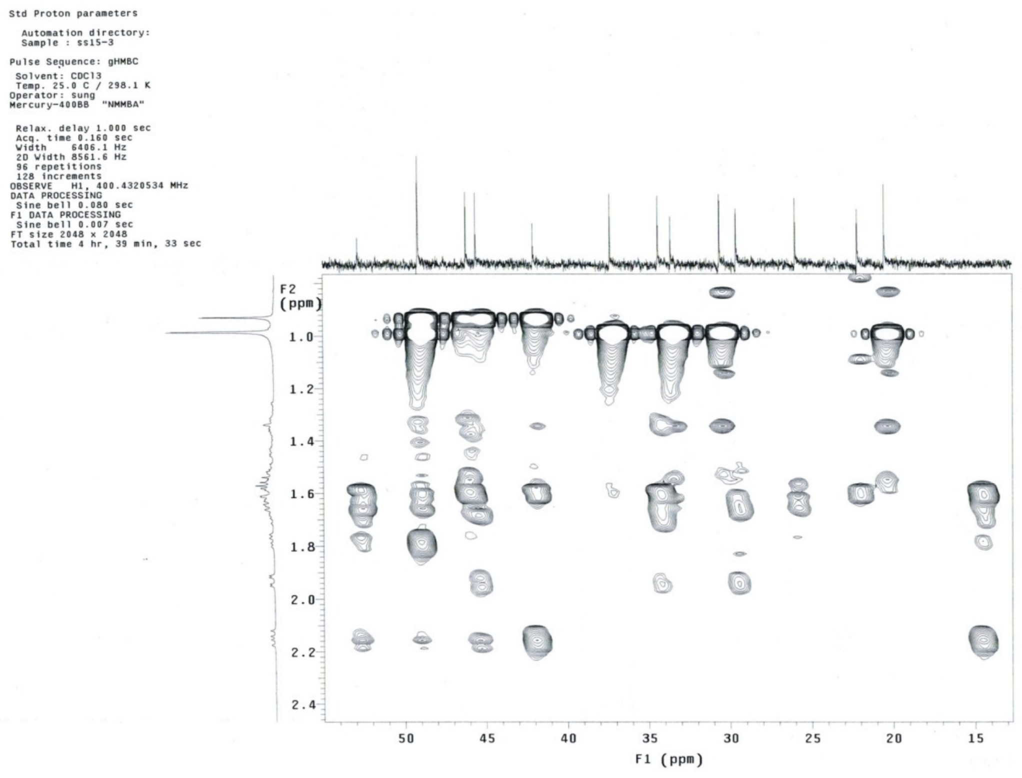

Figure S6. COSY spectrum (400 MHz) of compound **1** in $CDCl_3$.

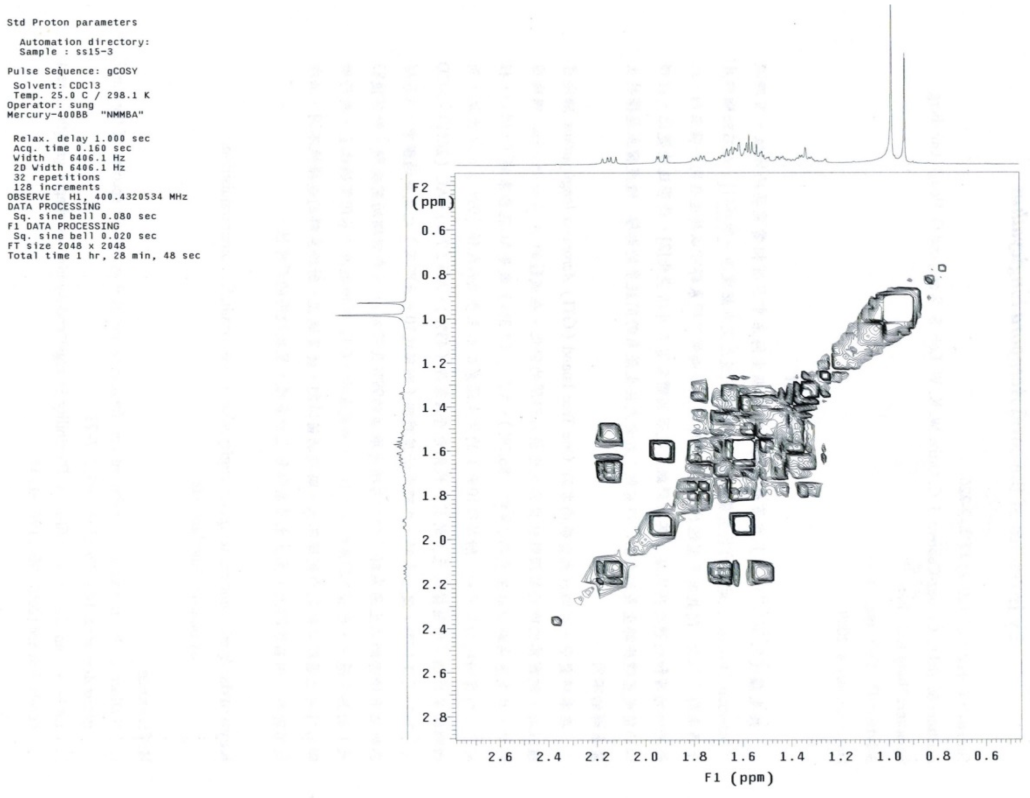

Figure S7. NOESY spectrum (400 MHz) of compound **1** in $CDCl_3$.

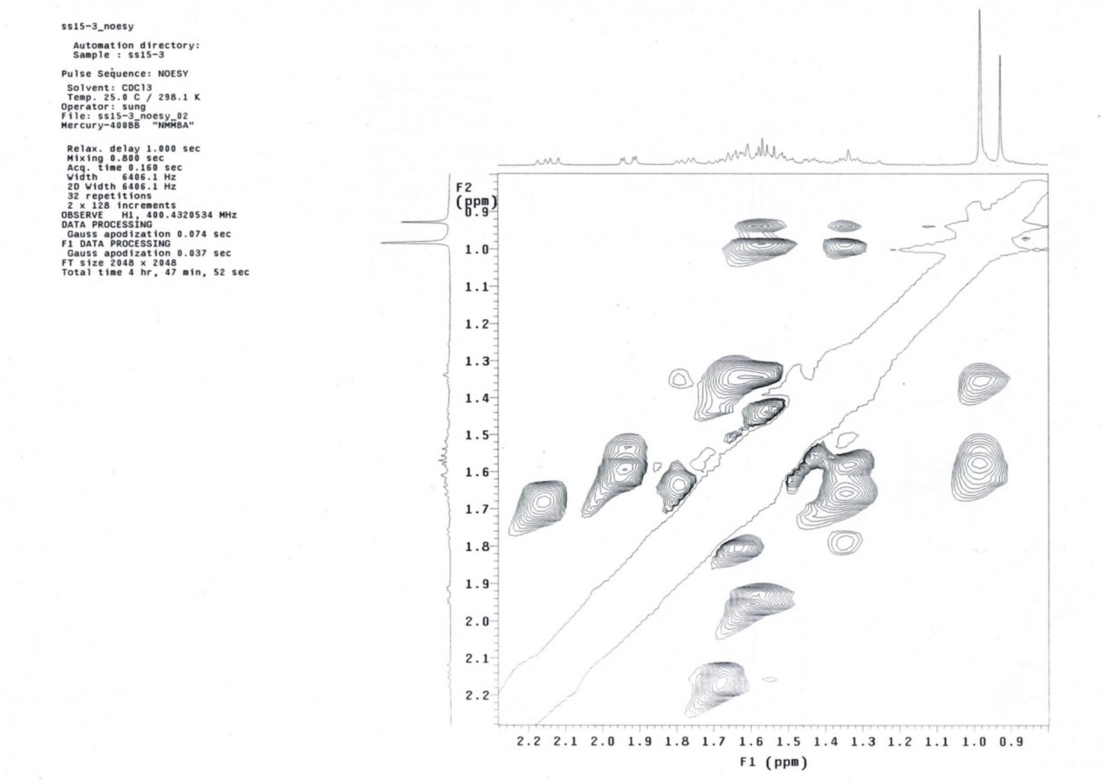

© 2014 by the authors; licensee MDPI, Basel, Switzerland. This article is an open access article distributed under the terms and conditions of the Creative Commons Attribution license (http://creativecommons.org/licenses/by/4.0/).
